# Bridges to treatment satisfaction: the roles of trauma, social support, race and ethnicity among perinatal women receiving behavioural activation therapy

**DOI:** 10.1186/s12916-025-04272-y

**Published:** 2025-08-20

**Authors:** Maya Zaidan, Andrea S. Lawson, Nicole Andrejek, Kate Walsh, Cindy-Lee Dennis, Samantha Meltzer-Brody, Richard K. Silver, Alison M. Stuebe, Simone N. Vigod, Daisy R. Singla

**Affiliations:** 1 Lunenfeld-Tanenbaum Research Institute, Sinai Health, Toronto, Canada; 2 Campbell Family Mental Health Research Institute, Centre for Addiction and Mental Health, Toronto, Canada; 3 Department of Psychology, Gender & Women’s Studies, University of Wisconsin-Madison, Madison, WI USA; 4 Department of Psychiatry, Temerty Faculty of Medicine, University of Toronto, Toronto, Canada; 5 Lawrence S. Bloomberg Faculty of Nursing, University of Toronto, Toronto, Canada; 6 Department of Psychiatry, School of Medicine, University of North Carolina, NC Chapel Hill, USA; 7 Department of Obstetrics & Gynecology, Endeavor Health (Formerly Northshore University HealthSystem), Evanston, USA; 8 Department of Obstetrics & Gynecology, University of Chicago Pritzker School of Medicine, Chicago, USA; 9 Department of Obstetrics and Gynecology, School of Medicine, University of North Carolina, NC Chapel Hill, USA; 10 Department of Psychiatry, Women’s College Hospital, Toronto, Canada

**Keywords:** Behavioural activation, Treatment satisfaction, Trauma, Social support, Race and ethnicity

## Abstract

**Background:**

High treatment satisfaction is related to improved treatment adherence and outcomes in psychotherapy research. Satisfaction with psychotherapy treatment among racially and ethnically diverse perinatal populations with post-traumatic stress (PTS) remains understudied. The aims of this study are to examine the relations between PTS symptoms, perceived social support, and race and ethnicity, and treatment satisfaction among perinatal women receiving behavioural activation (BA) psychotherapy.

**Methods:**

This is a mixed-methods study of the Scaling Up Maternal Mental healthcare by Increasing access to Treatment (SUMMIT) trial—a large, multi-site, non-inferiority trial for perinatal women with depressive and anxiety symptoms (NCT04153864). A two-sample *t*-test compared baseline PTS symptoms, social support, and treatment satisfaction between participants from white and racial and ethnic minority groups. Hierarchical multiple linear regression examined whether PTS symptoms, perceived social support, race and ethnicity predicted treatment satisfaction. Content analysis of open-ended responses explored facilitators and modifications for improving treatment satisfaction across PTS symptom severity and race and ethnicity.

**Results:**

Of 1230 women, 1119 (90.98%) had ≥ 1 BA session. Compared to their white counterparts, baseline PTS symptom severity was higher (*t*(1087) = − 4.98; *p* < 0.001; 95% CI = − 2.23, − 0.97), and social support lower (*t*(1087) = 8.05; *p* < 0.001; 95% CI = 0.43, 0.71) among racial and ethnic minority women. Lower baseline PTS symptom severity (*β* = − 0.009; 95% CI = − 0.016, − 0.002) and higher perceived social support (*β* = 0.042; 95% CI = 0.013, 0.072) were associated with higher post-treatment satisfaction across the sample. Descriptive analysis revealed no differences in treatment satisfaction across race and ethnicity; treatment satisfaction was higher for racial and ethnic minority women when social support was added to the regression model (*β* = 0.077; 95% CI = 0.005, 0.149). Content analysis (*n* = 807) revealed no differences by PTS symptoms severity or race and ethnicity across reported facilitators and modifications. BA as a treatment modality (*n* = 446, 55.27%) was a key facilitator; modifications included more sessions (*n* = 202, 25.03%).

**Conclusions:**

PTS symptom severity and social support predict treatment satisfaction among racially and ethnically diverse perinatal populations and should be considered when delivering psychotherapy.

**Trial registration:**

ClinicalTrials.gov NCT04153864. Registered on November 6, 2019.

**Supplementary Information:**

The online version contains supplementary material available at 10.1186/s12916-025-04272-y.

## Background

Treatment effectiveness is strongly related to treatment satisfaction, such that patients with higher treatment satisfaction are more likely to have higher adherence to treatment, better treatment outcomes, and a better quality of life than their less satisfied counterparts [1–3]. Individuals experiencing post-traumatic stress (PTS) symptoms are less satisfied with mental health treatment due to fears of stigma and shame while seeking mental healthcare [4]. This may be particularly true for perinatal (pregnant and up to one year postpartum) populations from racial and ethnic minority groups [5, 6], i.e. racially and ethnically underrepresented and marginalised individuals [7] who perceive accessing and engaging in mental health treatment as a negative experience due to the structural and discriminatory barriers, social stigma, trauma history, and a lack of cultural sensitivity on the part of the provider [8, 9]. In addition, these populations experience greater PTS symptom severity [5, 10], worse health outcomes, and poorer patient care [11]. As such, race and ethnicity are important determinants of health care access, as they capture broad systemic inequities in healthcare [11–13].

PTS symptoms during the perinatal period are common—approximately 8 million perinatal individuals worldwide develop post-traumatic stress disorder annually [14, 15]. Despite its frequent comorbidity with perinatal depressive and anxiety symptoms [14, 16, 17], perinatal PTS remains understudied [18–21]. Further, PTS symptoms [16, 17, 22] are associated with a high risk for adverse outcomes for both mother and child [23, 24]. Psychological treatments have been shown to be effective for comorbid PTS and depression [25–27] and outcomes may vary depending on individual differences at the start of treatment (e.g. baseline symptom severity [28–30]). Research into treatment satisfaction and its predictors (e.g. social support [3, 31]) for individuals with comorbid perinatal PTS and depression—particularly those from racial and ethnic minority groups—is important for providing insights on how to tailor treatments to enhance their efficacy and improve patient outcomes [15, 19, 32].

### Behavioural activation treatment for PTS

Behavioural Activation (BA) is a brief, evidence-based psychotherapy treatment that aims to engage patients in enjoyable activities aligned with their values and life goals [33]. While typically suggested for depressive and anxiety symptoms [34–36], BA has also demonstrated promising results for treating PTS symptoms and its co-occurrence with depressive symptoms [26]. BA treatment is likely effective at relieving PTS symptoms as some sessions address avoidance, encouraging clients to re-engage in aspects of their lives that they may have circumvented due to their trauma [26]. Understanding the extent to which perinatal individuals from racial and ethnic minority groups with comorbid depressive and PTS symptoms are satisfied with their BA treatment may further speak to the generalisability of BA as a transdiagnostic [Singla DR, Berenbaum TS, Silver RK, Vigod SN, Li S, Anand A: Bridges to treatment satisfaction: the roles of trauma, social support, race and ethnicity among perinatal women receiving behavioural activation therapy, Unpublished.] and patient-centred treatment [37].

### The SUMMIT trial

The current study is a secondary analysis of the Scaling Up Maternal Mental healthcare by Increasing access to Treatment (SUMMIT) trial—a large, multi-site psychotherapy trial that demonstrated the non-inferiority of task sharing and telemedicine to the gold standard approach of specialist-delivered, in-person BA for perinatal populations [38]. Specifically, the SUMMIT trial found non-inferiority between provider (non-specialist provider vs. specialist provider) and modality (telemedicine vs. in-person) of BA delivery on depressive and anxiety symptoms at 3 months post-randomisation [38], with high treatment satisfaction and low dropout rates. The term ‘telemedicine’ is used broadly amongst diverse healthcare providers delivering care virtually through secure, video-conferencing platforms. This mixed methods study combines quantitative assessments of self-reported satisfaction with qualitative insights about potential modifications and facilitators. 

Specifically, this study aims to examine:

1. The extent to which post-treatment satisfaction, baseline PTS symptom severity, and baseline social support differ between perinatal women from white and racial and ethnic minority groups receiving BA treatment;
2. The extent to which baseline clinical and demographic factors such as PTS symptom severity, perceived social support, and race and ethnicity predict post-treatment satisfaction with BA; and
3. Emerging facilitators of and modifications for improving treatment satisfaction with BA among perinatal women with mild, moderate, and severe baseline PTS symptom scores, and across race and ethnicity.

In doing so, the overall objective is to enhance the contextual understanding of treatment satisfaction, particularly for people who are at an increased risk of depressive and/or PTS symptoms and who have historically not been effectively engaged with in treatment studies [39, 40].

## Methods

### Study design

This is a mixed-methods study using data from the SUMMIT trial [41]. SUMMIT aimed to assess the comparable effectiveness between two types of providers (non-specialist vs. specialists) and modalities (telemedicine vs. in-person) of a brief BA treatment for perinatal depressive and anxiety symptoms. Participants were randomised to one of four arms based on provider type (specialist (e.g. psychiatrist, psychologist) vs. non-specialist (e.g. nurse, midwife, doula)) and treatment modality (in-person vs. telemedicine). All participants received the same BA treatment of 6–8 sessions (the SUMMIT BA manual is open access and freely available on the SUMMIT website [42]). The primary findings of the SUMMIT trial demonstrated non-inferiority between providers and modality on a range of outcomes, including depressive and anxiety symptoms as well as treatment satisfaction levels [38].

### Ethical approvals

The study received ethical approvals from the following three institutional review boards (IRB) for clinics in Chapel Hill, Chicago, and Toronto: University of North Carolina Biomedical IRB (19–1786); Endeavor Health IRB (EH18-129); and Clinical Trials Ontario (1895). No additional ethics approval or informed consent was required for this secondary analysis.

### Study participants

Inclusion criteria for the larger SUMMIT trial included individuals who were pregnant (up to 36 weeks) or postpartum (4–30 weeks), aged ≥ 18, spoke English or (US sites) Spanish, and had an Edinburgh Postnatal Depression Scale (EPDS) [43] score of ≥ 10. Exclusion criteria included active suicidal intent, psychosis, mania, substance abuse, ongoing psychotherapy, recent psychotropic medication use, severe foetal anomalies, and stillbirth or infant death during the index pregnancy.

The current study included a subset of participants from the SUMMIT trial who received at least one BA treatment session in order to assess treatment satisfaction. The term ‘women’ is used throughout, and includes participants who identified as genderqueer/gender non-conforming, different identity, or who preferred not to answer.

### Data collection

Participants were recruited from hospitals and clinics in Chapel Hill, North Carolina; Chicago, Illinois; and Toronto, Canada [41]. A trained research assistant or clinical provider introduced a potential participant to the study, and informed consent was obtained in person or remotely via the Research Electronic Data Capture (REDCap™) system. Eligible participants completed baseline measures, including demographics and clinical assessments (e.g. depressive/PTS symptoms). The data analysed for this study included assessments measured at baseline to 3 months post-randomisation.

### Measures

Baseline variables included self-reported age, perinatal period (antenatal vs. postnatal), recruitment location, race and ethnicity, gender identity, marital status, education, employment status, household income, and immigration status. Race and ethnicity was categorised as ‘white’ for those identifying as white and European, and ‘racial and ethnic minority’ for those identifying with one of the following racial and ethnic groups: Asian; Black; First Nations/Aboriginal; Hawaiian/Pacific Islander; Hispanic; Middle Eastern; or Mixed race. For our main analyses, racial and ethnic minority groups were combined due to their shared experience of systemic discrimination and marginalisation [44]. Future research should explore subgroup differences to better capture the diversity within these groups.

The Edinburgh Postnatal Depression Scale (EPDS) [43] is a 10-item self-report Likert scale with summed scores ranging from 0 to 30 (0 = not at all, 3 = most of the time). The EPDS measures levels of depressive symptoms during the perinatal period, with higher scores indicating higher levels of depressive symptoms. The EPDS is a validated screening tool for use during the perinatal period [45] and has good sensitivity, specificity, and sensitivity to change [43].

PTS symptom severity was assessed using the Abbreviated PTSD Checklist (PCL-6) [46]. This is a 6-item Likert scale, with summed scores ranging from 6 to 30 (1 = ‘not at all’; 5 = ‘extremely’). The PCL-6 has been used with perinatal populations [47, 48] and has good psychometric properties [49]. We used a cut-off score of ≥ 14 to indicate a clinical threshold for PTS [46], and divided the sample into three groups based on symptom severity: mild (PCL-6: 6-13), moderate (PCL-6: 14-20), and severe (PCL-6: 21-30).

Participants’ perceptions of social support (i.e. friends, family, significant others) were assessed using the Multidimensional Scale of Perceived Social Support (MSPSS) [50]. This is a 12-item Likert scale (1 = very strongly disagree, 7 = very strongly agree) uses mean scores ranging from 1 to 7, with higher scores reflecting higher perceived social support. The questionnaire has been used in perinatal populations previously [51, 52] and has high internal reliability and validity [53, 54]. The EPDS, PCL-6 and MSPSS were completed at baseline.

Treatment satisfaction was assessed using the Client Satisfaction Questionnaire (CSQ-8) [55] at 3-months post-randomisation. The CSQ-8 is an 8-item tool with scores rated on a 4-point Likert scale (1 = low satisfaction, 4 = high satisfaction). The CSQ-8 has been used in perinatal populations previously [37] and has high internal reliability and validity across mental health settings [56, 57] and good psychometric properties with high internal consistency [55]. Two open-ended questions were added to the CSQ-8: [1] ‘What did you like about the therapy sessions?’ and [2] ‘What would you change about the therapy sessions?’.

### Statistical analysis

Baseline variables were assessed by estimating means and either 95% confidence intervals (CIs) for continuous variables or frequencies for categorical variables.

A two-sample *t*-test was conducted to examine differences in post-treatment satisfaction (CSQ-8), baseline PTS symptom severity (PCL-6) and baseline social support (MSPSS) between the white and racialized groups. An ANOVA was conducted to compare the differences in treatment satisfaction across all racial and ethnic subgroups. Additional subgroup analyses of clinical variables were conducted to investigate potential subgroup differences (see Additional File 1: Table 4).

Using hierarchical multiple linear regression, we examined whether baseline PTS symptom severity, race and ethnicity, and perceived social support predicted post-BA treatment satisfaction. Predictors were added sequentially to assess their individual effects on post-treatment satisfaction. In Model 1, control variables were added, including age, perinatal period, education level, employment status, immigration status, provider type, delivery type, treatment dosage (i.e. number of treatment sessions), late completion of follow up assessments, and depressive symptoms. Baseline PTS symptom severity (Model 2), race and ethnicity (Model 3), and baseline perceived social support (Model 4) were added sequentially in each subsequent model. The selection of predictors (i.e. PTS, race and ethnicity, and social support) was based on previous literature (e.g. [3, 11, 13, 16, 18, 31]). The selection of covariates, including demographic (age, education, immigration status, employment status) and clinical (depressive symptoms, treatment dosage, perinatal period, provider type, treatment delivery and late completion of satisfaction survey) variables, was made because they may influence treatment satisfaction [58–61] and mitigate any potential confounders and retrospective bias. Prior to analysis, assumption checks for independence of residuals, linearity, normality, and homoscedasticity were conducted and satisfied. No influential/leverage points or multicollinearity was found. Significance was defined as *p*-value ≤ 0.05. Missing data were imputed using multiple imputation by an independent biostatistician who used fully conditional specification (FCS) methods in SAS Proc MI and Proc MIANALYZE. All other statistical analyses were conducted in SPSS. We used a pairwise deletion of the total eligible sample to avoid excluding those who preferred not to disclose their race and ethnicity, education level, immigration status, or employment status (*n* = 43; 3.8% of total sample), allowing their available data in other variables to be retained in the analyses.

### Qualitative data analysis

Participants received the CSQ-8 at 3 months post-randomisation. Once participants responded to the CSQ-8 survey questions, they were asked to fill out two open-ended questions. These questions were [1] ‘What did you like about the therapy sessions?’ and [2] ‘What would you change about the therapy sessions?’. A subsample of participants who completed the CSQ-8 also responded to the two open-ended questions (*n* = 807, 72.12%). Answers were collated and coded inductively using Microsoft Excel. A trained research assistant conducted a content analysis of the data using previously established methodologies [62]. Themes emerged inductively, meaning that as participant responses were coded, they were subsequently organised into commonly endorsed themes. Interrater reliability was calculated using Kappa’s (*κ*) coefficient between two independent raters and deemed strong (*κ* = 0.89) [63]. Emerging facilitators (responses to Question 1) of and modifications (responses to Question 2) for improving treatment satisfaction endorsed by > 10% of the total sample were extracted and reported. Although less endorsed themes were identified, they were eventually collapsed due to low individual frequency and high conceptual overlap. As such, broader themes were reported to emphasise the most salient themes in the data. The sample was then split into three groups based on PTS symptom severity levels: mild (6-13), moderate (14-20), and severe (21-30), and further between racial and ethnic groups to examine potential descriptive differences between groups in an effort to identify specific group needs at baseline that may be intentionally incorporated throughout treatment.

## Results

**Fig. 1:**
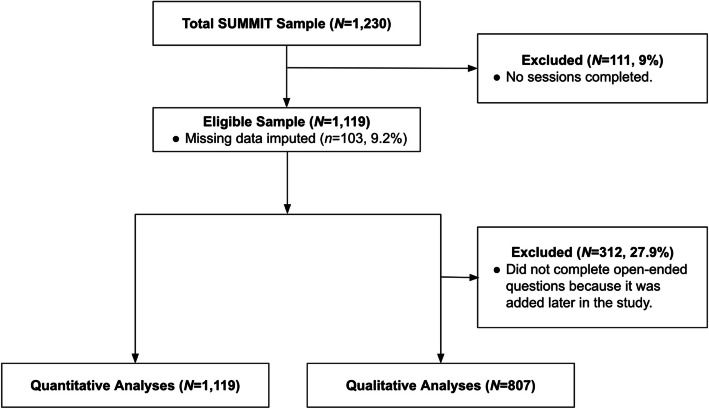
Participant flow chart

Between January 2020 through October 2023, *N* = 1119 women were enrolled, randomised, and received ≥ 1 BA session. The overall sample (*N* = 1119) and qualitative sub-sample (*N* = 807) showed similar demographic and clinical characteristics (Fig. 1; see Additional File 2: Table 5).

Baseline sociodemographic and clinical characteristics are described in Table 1. In short, participants were 33.35 years of age (95% CI 33.06–33.64), half were postpartum (*n* = 563, 50.31%), and almost half (*n* = 526, 47.01%) were from racial and ethnic minority groups (for clinical characteristics of racial and ethnic subgroups see Additional File 1: Table 4). The vast majority identified as cis-women (*n* = 1079, 96.43%). At baseline, participants reported moderate symptoms for both PTS (PCL-6 = 16.90, 95% CI 16.59–17.22) and depression (EPDS = 15.75, 95% CI 15.52–15.97), and high perceived social support (MSPSS = 5.31, 95% CI 5.24–5.38). They attended a mean number of 6.82 out of 8 BA sessions (95% CI 6.70–6.94) and reported high post-treatment satisfaction (CSQ-8 = 3.40 out of 4, 95% CI 3.37–3.44).

**Table 1:** Baseline characteristics of participants (*N* = 1119), *N* (%) unless otherwise indicated

**Characteristic**	**Sample (*N*=1,119)**
**Demographic variables (baseline)**	
Age, *mean (95% CI)*	33.35 (33.06, 33.64)
Perinatal period at first session	
Pregnant	556 (49.69)
Location	
Canada	751 (67.11)
United States	368 (32.89)
Race and ethnicity recategorized	
Racial and ethnic minority group*	526 (47.01)
White	563 (50.31)
Prefer not to answer	30 (2.68)
Race and ethnicity by subgroup	
Asian	192 (17.16)
Black	113 (10.10)
First Nations/Aboriginal	5 (0.45)
Hawaiian/Pacific Islander	4 (0.36)
Hispanic	93 (8.31)
Middle Eastern	30 (2.68)
Mixed-race	89 (7.95)
White and European	563 (50.31)
Prefer not to answer	30 (2.68)
Gender identity	
Female	1079 (96.43)
Genderqueer/Gender non-conforming	2 (0.18)
Different identity	1 (0.09)
Prefer not to answer	1 (0.09)
Marital status	
Married or stable relationship	966 (86.33)
Single	139 (12.42)
Prefer not to answer	14 (1.25)
Education	
University (graduate and undergraduate)	799 (71.40)
College/Trade school	186 (16.62)
Highschool and below	125 (11.17)
Prefer not to answer	9 (0.80)
Employment	
Employed	565 (50.49)
Unemployed	552 (49.33)
Missing	2 (0.18)
Household income (based on postal code)	
$0-$39,999	307 (27.44)
$40,000-$79,999	640 (57.19)
$80,000+	92 (8.22)
Prefer not to answer	80 (7.15)
Immigration status (born in country of residence)	
Yes	779 (69.62)
No	334 (29.85)
Prefer not to answer	6 (0.54)
Provider type (specialist/non-specialist)	
Specialist	566 (50.58)
Delivery type (in-person/telemedicine)	
In-person	232 (20.73)
**Clinical variables (baseline), ***mean (95% CI)*	
Post-traumatic stress symptoms, PCL-6^a^	16.90 (16.59, 17.22)
Depression symptoms, EPDS^b^	15.75 (15.52, 15.97)
Perceived social support, MSPSS^c^	5.31 (5.24, 5.38)
**Process variables (post-treatment), ***mean (95% CI)*	
Treatment Satisfaction at 3-months post-treatment, CSQ-8^d^	3.40 (3.37, 3.44)
Treatment dosage (no. of completed sessions)	6. 82 (6.70, 6.94)
Late completion of 3-month questionnaire, *n(%)*	6 (0.54)

*Racial and ethnic minority groups= Asian, Black, First Nations/Aboriginal, Hawaiian/Pacific Islander, Hispanic, Middle Eastern and Mixed race

^a^Abbreviated PTSD Checklist-6, scored from 6-30

^b^Edinburgh Postnatal Depression Scale, scored from 0-30

^c^Multidimensional Scale of Perceived Social Support, scored from 1-7

^d^Client Satisfaction Questionnaire, scored from 0-4

### Treatment satisfaction, PTS and social support across racial and ethnic groups

**Fig. 2:**
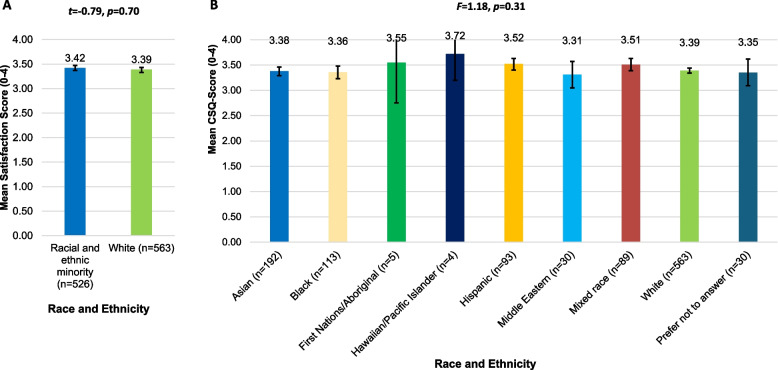
Post-treatment satisfaction scores (CSQ-8) across racial and ethnic groups (*N* = 1119). **A** Racial and ethnic minority groups and white group. **B** Race and ethnicity subgroups

Compared to their white counterparts, racial and ethnic minority women reported statistically significantly higher baseline PTS symptom severity scores (PCL-6 = 16.13 (95% CI 15.72–16.54) vs. 17.73 (95% CI 17.25–18.21), *t*(1087) = − 4.98, *p* < 0.001, 95% CI = − 2.23, − 0.97) and statistically lower social support scores (MSPSS = 5.59 (95% CI 5.50–5.67) vs. 5.01 (95% CI 4.90–5.13), *t* = 8.05(1087), *p* < 0.001, 95% CI = 0.43, 0.71). Post-treatment satisfaction scores were similarly high between the white and racial and ethnic minority women (CSQ = 3.39 (95% CI 3.34–3.44) vs. 3.42 (95% CI 3.37–3.47); *t*(1087) = − 0.79, *p* = 0.43, 95% CI = − 0.10, 0.04) (see Fig. 2A) and there were no differences within racial and ethnic subgroups (*F*(8, 1110) = 1.18, *p* = 0.31) (see Fig. 2B).

### Predictors of treatment satisfaction

#### Model 1

In the first model, we examined the control variables (age, perinatal period, education level, employment status, immigration status, provider type, delivery type, treatment dosage, late completion of follow-up assessments and depressive symptoms). We found that higher educational attainment (*β* = − 0.05, *t* = − 2.64, *p* = 0.08) and more BA sessions attended (*β* = − 0.13, *t* = 15.75, *p* < 0.001) were significant predictors of post-treatment satisfaction. The overall model was statistically significant, *F*(10, 1072) = 25.58, *p* < 0.001, and accounted for 18.5% of the variance in post-treatment satisfaction.

#### Model 2

In the second model, we added PTS symptom severity to model 1. We found that lower baseline PTS symptom severity significantly predicted higher post-treatment satisfaction scores (*β* = − 0.01, *t* = − 2.80, *p* = 0.05). The overall model was statistically significant, *F*(11, 1071) = 24.12, *p* < 0.001, and accounted for 19% of the variance in post-treatment satisfaction.

#### Model 3

In the third model, we added race and ethnicity to model 2, but race and ethnicity did not significantly predict post-treatment satisfaction (*β* = 0.06,* t* = 1.66, *p* = 0.10). The overall model was statistically significant, *F*(12, 1070) = 22.30, *p* < 0.001, and accounted for 19.2% of the variance in post-treatment satisfaction.

#### Model 4

In our final model, we added perceived social support at baseline to model 3. We found that perceived social support at baseline positively predicted post-treatment satisfaction (*β* = 0.04, *t* = 2.84, *p* = 0.05). Race and ethnicity significantly predicted treatment satisfaction (*β* = 0.08, *t* = 2.10, *p* = 0.04) such that racial and ethnic minority women had higher post-treatment satisfaction scores than white women. Similar to the previous models, lower baseline PTS symptom severity (*β* = − 0.01, *t* = − 2.48, *p* = 0.01), higher education levels (*β* = − 0.06, *t* = − 3.06, *p* = 0.002) and higher number of attended sessions (*β* = 0.13, *t* = 15.61, *p* < 0.001) remained significant predictors of treatment satisfaction. The overall model was statistically significant, *F*(13, 1069) = 21.41, *p* < 0.001, accounting for 19.7% of the variance in post-treatment satisfaction.

For a summary of all four models see Table 2.

**Table 2:** Hierarchical multiple linear regression predicting treatment satisfaction (*N* = 1119)

Treatment satisfaction	Model 1 Covariates only	Model 2 (M1 + PTS symptoms)	Model 3 (M2 + race and ethnicity)	Model 4 (M3 + social support)
	*β *(95% CI)	*β *(95% CI)	*β *(95% CI)	*β *(95% CI)
Constant	2.832 (2.533, 3.132)***	2.952 (2.641, 3.262)***	2.887 (2.567, 3.206)***	2.623 (2.256, 2.989)***
Age	− 0.002 (− 0.009, 0.006)	− 0.002 (− 0.009, 0.005)	− 0.002 (− 0.009, 0.005)	− 0.001 (− 0.008, 0.006)
Perinatal period at baseline (Postpartum)	0.008 (− 0.064, 0.081)	0.000 (− 0.072, 0.073)	0.001 (− 0.071, 0.074)	0.003 (− 0.069, 0.075)
Higher education	− 0.047 (− 0.082, − 0.012)**	− 0.052 (− 0.087, − 0.017)**	− 0.049 (− 0.084, − 0.014)**	− 0.055 (− 0.091, − 0.020)**
Employed	−.051 (− 0.023, 0.125)	0.040 (− 0.035, −.114)	0.044 (− 0.031, 0.118)	0.041 (− 0.033, 0.116)
Birthplace (resident)	− 0.024 (− 0.096, 0.049)	− 0.031 (− 0.104, 0.041)	− 0.008 (− 0.085, 0.069)	− 0.014 (− 0.091, 0.064)
Provider (specialist)	0.028 (− 0.037, 0.094)	0.028 (− 0.038, 0.093)	0.027 (− 0.038, 0.092)	0.025 (− 0.040, 0.090)
Treatment delivery (in-person)	0.035 (− 0.047, 0.117)	0.030 (− 0.051, 0.112)	0.028 (− 0.053, 0.110)	0.020 (− 0.062, 0.101)
Treatment dosage	0.130 (0.113, 0.146)***	0.129 (0.113, 0.146)***	0.130 (0.114, 0.146)***	0.128 (0.112, 0.144)***
Late completion	0.087 (− 0.361, 0.534)	0.068 (− 0.378, 0.514)	0.067 (− 0.378, 0.513)	0.046 (− 0.391, 0.484)
Depression symptoms^a^	− 0.007 (− 0.016, 0.002)	0.000 (− 0.010, 0.010)	0.000 (− 0.010, 0.009)	0.001 (− 0.008, 0.011)
PTS symptoms^a^	-	− 0.010 (− 0.017, − 0.003)**	− 0.011 (− 0.018, − 0.003)**	− 0.009 (− 0.016, − 0.002) **
Race and ethnicity (racial and ethnic minority group)	-	-	0.060 (− 0.011, 0.131)	0.077 (0.005, 0.149)**
Perceived social support^a^	-	-	-	0.042 (0.013, 0.072)**
Model fit	*R*^2^ =.193 *R*^2^_adjusted_ =.185***	*R*^2^ =.199 *R*^2^_adjusted_ =.190***	*R*^2^ =.201 *R*^2^_adjusted_ =.192	*R*^2^ =.207 *R*^2^_adjusted_ =.197***

Model = ‘Enter’ method in SPSS

^a^baseline measure; *β*, unstandardised regression coefficient; *CI*, confidence interval; *R*^2^, coefficient of determination; *R*^2^_adjusted_, adjusted* R*^2^; *PTS*, post-traumatic stress; *M1*, Model 1; *M2*, Model 2; *M3*, Model 3

*p < 0.05. **p < 0.01. ***p < 0.001

### Facilitators of and modifications for improving treatment satisfaction

For a summary of facilitators and modifications across PTS symptom severity groups and across race and ethnicity in the mild and severe PTS groups see Table 3. Some participants did not report any facilitators (*n* = 61, 7.56%) or modifications (*n* = 272, 33.71%) for improving treatment satisfaction. The most endorsed facilitators and desired modifications are detailed below.

**Table 3:** Reported facilitators and modifications for improving treatment satisfaction across post-traumatic symptom severity and race and ethnicity

Responses *n*(%)	All	Mild (PCL-6: 6-13)*			Moderate (PCL-6: 14-20)	Severe (PCL-6: 21-30)*		
	**Total** **(*N* = 807)**	**Racial and ethnic minority** **(*n* = 94)**	**White** **(*n* = 119)**	**Total** **(*N* = 219)**	**Total** ***(N* = 378)**	**Racial and ethnic minority** **(*n* = 110)**	**White** **(*n* = 95)**	**Total** **(*N* = 210)**
**Facilitators**								
(1) BA as a treatment modality	446 (55.27)	45 (47.87)	70 (58.82)	118 (53.88)	224 (59.26)	44 (40.00)	60 (63.16)	104 (49.52)
(2) Treatment provider	381 (47.21)	49 (52.13)	57 (47.90)	110 (50.23)	175 (46.30)	44 (40.00)	50 (52.63)	96 (45.71)
(3) Individual talk therapy	174 (21.56)	18 (19.15)	25 (21.01)	45 (20.55)	75 (19.84)	28 (25.45)	25 (26.32)	54 (25.71)
No facilitators reported	61 (7.56)	8 (8.51)	10 (8.40)	18 (8.22)	20 (5.29)	20 (18.18)	2 (2.11)	23 (10.95)
**Suggested modifications**								
(1) More sessions	202 (25.03)	12 (12.77)	19 (15.97)	32 (14.61)	105 (27.78)	34 (30.91)	31 (32.63)	65 (30.95)
(2) Flexibility with scheduling sessions	111 (13.75)	15 (15.96)	17 (14.29)	33 (15.07)	54 (14.29)	15 (13.64)	7 (7.37)	24 (11.43)
No modifications suggested	272 (33.71)	47 (50.00)	39 (32.77)	89 (40.64)	107 (28.31)	42 (38.18)	32 (33.68)	76 (36.19)

*BA* behavioural activation

*Mild and severe groups were further separated by race and ethnicity to distinctly explore differences on each side of the spectrum

#### Facilitator 1: BA as a treatment modality

The most commonly endorsed facilitator of treatment satisfaction was BA as a treatment modality (*n* = 446, 55.27%). Participants expressed appreciation for various aspects of BA, including its content, tools, structure, and activities. Participants also reported specific benefits, such as motivation towards action, enhanced self-accountability, the acquisition of new strategies and perspectives to help themselves, and general emotional benefits. This facilitator was highly endorsed among both white and racial and ethnic minority women; however, this facilitator was more commonly endorsed by white women in the mild (white: *n* = 70, 58.82%, racial and ethnic minority: *n* = 45, 47.87%) and severe PTS groups (white: *n* = 60, 63.16%, racial and ethnic minority: *n* = 44, 40%).

*"Activities were practical with [my] current home life situation. Some of the prompts like implementing values in everyday life, encouraged me to take control of my activities and helped me feel better."*(201_severe PTS_racial and ethnic minority)

#### Facilitator 2: treatment provider

The second most commonly endorsed facilitator of treatment satisfaction was the treatment provider (*n* = 381, 47.21%). Participants expressed general appreciation for their treatment provider or commended their provider’s interpersonal skills, patient-centredness, knowledge, and cultural sensitivity. Variation in endorsement was found in the severe PTS group (white: *n* = 50, 52.63%, racial and ethnic minority: *n* = 44, 40%).

*"[Treatment provider] was warm, accessible, and insightful...For me, the most effective part of the therapy was talking to [provider’s name] and doing check-ins."* (630_severe PTS_ racial and ethnic minority)

*"I liked that the provider seemed to work from an anti-discrimination and anti-racist perspective [...] was very evident in the manner the provider spoke. As a person of colour [POC] that is what really allowed me to buy into the treatment."* (383_severe PTS_ racial and ethnic minority)

#### Facilitator 3: features of individual talk therapy

The third most commonly endorsed facilitator of treatment satisfaction was general elements of individual talk therapy (*n* = 174, 21.56%). Participants like regular, 1:1 sessions with someone to talk to in a non-judgmental and unbiased capacity. There were no group differences in the endorsement in this facilitator.

*"Having a regular check-in/touchpoint where I focused on me and how I was feeling [...] It really hit home that simply drawing awareness to what I was doing helped to pinpoint when I felt good and when I didn’t."* (329_moderate PTS_ racial and ethnic minority)

#### Suggested modification 1: more sessions

The most endorsed suggested modification across participants was the desire for more sessions (*n* = 202, 25.03%). Participants suggested more or longer sessions, follow-up sessions, the ability to continue with their treatment provider, and to be referred to more specialist care or given other resources after their last session. Irrespective of race and ethnicity, this suggested modification was most commonly endorsed by the moderate (*n* = 105, 27.78%) and severe (*n* = 65, 30.95%) PTS groups, with the lowest endorsement rates in the mild PTS group (*n* = 32, 14.61%).

*"I would like more time, the 8 weeks seemed too short and I was really feeling like I was starting to get somewhere [...]"* (081_severe PTS_ racial and ethnic minority)

#### Suggested modification 2: flexibility with scheduling sessions

The second most endorsed modification was increased flexibility in scheduling sessions (*n* = 111, 13.75%). Some participants noted difficulty of a fixed timeslot and location when motherhood is unpredictable. This suggested modification was endorsed similarly across all groups.

*"Having more flexibility for session days. I often had to reschedule and the therapist was limited by [their] set days."* (391_mild PTS_white)

## Discussion

In this mixed-methods secondary analysis of the SUMMIT trial, we examined: (1) differences in post-treatment satisfaction, baseline PTS symptom severity and perceived social support among white and racial and ethnic minority women; (2) whether baseline PTS symptom severity, perceived social support and race and ethnicity, predict post-treatment satisfaction; and (3) suggested facilitators of and modifications for brief, BA psychotherapy. By integrating quantitative and qualitative findings, we aimed to provide a nuanced and holistic understanding of treatment satisfaction among a racially and ethnically diverse sample of perinatal women. The results of this study are explored in relation to the wider literature below.

### Post-traumatic stress (PTS) and treatment satisfaction

As demonstrated by others [59, 64], higher PTS symptom severity scores at baseline were negatively correlated with post-treatment satisfaction scores, even when controlling for comorbid depressive symptoms. Given the high comorbidity of PTS symptoms with depression [4–6], this finding illustrates the role of PTS symptom severity as a predictor of BA satisfaction. Additionally, we found that other variables such as race and ethnicity, baseline perceived social support, education level, and treatment dosage were also significant predictors of post-treatment satisfaction alongside PTS symptom severity. Taken together, these findings emphasise the importance of PTS symptom severity while also recognising the complex interplay and diverse range of factors such as perceived social support that can impact treatment satisfaction. We recommend healthcare providers screen for PTS symptoms concurrently with depressive or anxiety symptoms irrespective of their clinical severity on these scales given their high comorbidity. PTS symptom scores of 14 [49] or above may suggest the need to focus treatment on addressing trauma first given its predictive ability in treatment satisfaction. Future studies should investigate whether these findings are similar for other treatment modalities and clinical populations beyond BA and perinatal populations with comorbid PTS and depressive symptoms.

### The importance of social support

Similar to others [3, 31, 65], we also found perinatal participants with higher levels of perceived social support at baseline had higher satisfaction scores. Importantly, in low and middle-income countries, perceived support from one’s significant other, extended family, and community members has been shown as a consistent mediator of brief psychological treatments in improving mental health outcomes among perinatal populations [66, 67]. Thus, perceived social support may serve as either a protective or risk factor for perinatal populations. Further research should study how to bolster perceived support in psychological treatments, including the potential inclusion of significant others [9] to allow for more family-centred care beyond the inclusion of mother and child alone.

Potentially related to social support is the therapeutic relationship between the participant and provider [68], which was highlighted as the second most endorsed facilitator of participants’ experience with treatment irrespective of treatment provider*.* Specifically, women reported that non-specific therapy skills such as their treatment provider’s ability to create a safe and supportive environment facilitated their satisfaction with treatment. Skar-Fröding and colleagues [3] showed that those who felt well supported by mental health staff on their personal recovery journeys were also more satisfied with treatment. As such, a good therapeutic alliance may facilitate treatment satisfaction [11, 69–71]. Collectively, these mixed-method findings illustrate the multifaceted layers of social support and suggest that increased support from friends, family, or their healthcare provider influences a participant’s experience and satisfaction with psychotherapy.

### High treatment satisfaction scores among racial and ethnic minority women

Interestingly, we found that women from racial and ethnic minority groups were more likely to be satisfied with treatment than women who identified as white when baseline perceived social support was added to the model. This was an unexpected finding given the well-documented healthcare disparities experienced by individuals from racial and ethnic minority groups as a result of systemic racism and discrimination. Specifically, previous studies have demonstrated that these inequities contribute to more negative experiences accessing and engaging in mental health treatment [15, 16, 72], a reduced likelihood of receiving adequate mental healthcare [73, 74] and an increased treatment dropout [75, 76] all of which are suggestive of low treatment satisfaction. While there was a statistically significant difference in baseline PTS symptoms between white and racial and ethnic minority women, this difference did not exceed the minimal clinically important difference established for the PCL-6 [49], suggesting that baseline PTS symptoms alone does not fully explain the observed differences in treatment satisfaction. A plausible explanation for our finding is the culturally sensitive psychotherapy provided in the SUMMIT trial [37]—defined as the willingness and ability to adapt an interpersonal stance that is curious, patient-centred and culturally-aware [77, 78]. In the SUMMIT trial, this means that topics regarding race, ethnicity and culture were explicitly addressed during treatment and supervision through the lens of cultural humility, cultural comfort and cultural opportunities [37]. It is possible that SUMMIT provided the needed perceived support and a satisfying therapeutic experience for women from racial and ethnic minority backgrounds, who typically face systemic barriers and inequities in access to psychotherapy [15, 16, 79], compared to their white counterparts. Furthermore, the qualitative analyses showed that 'BA as a treatment modality' and 'features of individual talk therapy' were commonly endorsed as a facilitator across both racial and ethnic groups, as was the desire for more sessions. These qualitative findings suggest that the BA intervention was perceived as therapeutic and helpful, irrespective of the participant’s race and ethnicity. For additional details related to the culturally-sensitive care offered in the SUMMIT trial, please refer to published manuscripts [37] and [80].

### Strengths and limitations

To our knowledge, this is among the first examinations of PTS symptom severity, perceived social support, race and ethnicity, and treatment satisfaction in perinatal women. Strengths of this study include a large, geographically and racially diverse sample, high treatment satisfaction rates, and the use of open-ended qualitative data to better understand the experience of receiving therapy. Despite the strengths of the current study, there are also some limitations. First, large differences in sample sizes did not permit sufficiently powered analyses between certain racial and ethnic groups [81]. Thus, information regarding intergroup differences may be missing, resulting in less specificity. Despite this, it was imperative to include all racial and ethnic subgroups irrespective of sample size to avoid reinforcing systematic patterns of exclusion in research [82]. Furthermore, the grouping is theoretically relevant to the study in describing general racialised experiences, and although the sample sizes are regionally representative, future studies may wish to compare various racial and ethnic groups (see Additional File 1: Table 4). Second, we chose the 6-item PCL-6 as our measure of PTS symptoms in an effort to be patient-centred given the scale’s brevity, in contrast to the twenty-item PCL-5 [83]. However, the PCL-5 is the most used DSM-5-based iteration of the PCL scales; thus, our results may not be generalisable to studies using the PCL-5. Third, qualitative results were collected using two optional open-ended questions. In future, data could be obtained systematically through semi-structured interviews.

## Conclusions

Overall, our study highlights the role of diverse factors such as PTS symptom severity, perceived social support, race and ethnicity, and the treatment and its provider in influencing satisfaction with BA treatment among perinatal women with comorbid depressive and PTS symptoms. In addition, our results suggest that brief psychotherapies, such as BA, can be delivered and received from a culturally-sensitive lens. By considering these factors, we can enhance the patient-centredness and effectiveness of psychotherapies for perinatal populations globally.

## Supplementary Information

Additional File 1: Table 4. Clinical characteristics by race and ethnicity subgroups including means, 95% confidence intervals, and comparison statistics using an ANOVA, including Welch’s ANOVA and post-hoc comparisons. Clinical characteristics assessed in this table include: Age, post-traumatic stress symptoms, depression symptoms, perceived social support, treatment satisfaction at 3-months, and treatment dosage. The racial and ethnic subgroups include: Asian, Black, First Nation/Aboriginal, Hawaiian/Pacific Islander, Hispanic, Middle eastern, Mixed Race, White, and Prefer not to answer

Additional File 2: Table 5. Baseline characteristicsof the overall sample and the qualitative sub-sample

## Data Availability

Two years after publication, all individual participant de-identified data and analytic code will be shared with researchers who submit a methodologically sound proposal to: summittrial@sinaihealth.ca. Data will be available indefinitely.

## Abbreviations

BA: Behavioural activation
CIs: Confidence intervals
CSQ-8: Client Satisfaction Questionnaire
EPDS: Edinburgh Postnatal Depression Scale
FCS: Fully conditional specification
IRB: Institutional Review Boards
MSPSS: Multidimensional Scale of Perceived Social Support
PCL-6: Abbreviated PTSD checklist
PTS: Post-traumatic stress
SUMMIT: *S*caling *U*p *M*aternal *M*ental healthcare by *I*ncreasing access to *T*reatment
