# Supplementary material for: Bridges to treatment satisfaction: the roles of trauma, social support, race and ethnicity among perinatal women receiving behavioural activation therapy
Source: BMC Med. 2025 Aug 20;23:489. doi: 10.1186/s12916-025-04272-y (PMC12366383; doi:10.1186/s12916-025-04272-y)
Supplement: Supplementary file 1 — Additional File 1: Table 4. Clinical characteristics by race and ethnicity subgroups including means, 95% confidence intervals, and comparison statistics using an ANOVA, including Welch’s ANOVA and post-hoc comparisons. Clinical characteristics assessed in this table include: Age, post-traumatic stress symptoms, depression symptoms, perceived social support, treatment satisfaction at 3-months, and treatment dosage. The racial and ethnic subgroups include: Asian, Black, First Nation/Aboriginal, Hawaiian/Pacific Islander, Hispanic, Middle eastern, Mixed Race, White, and Prefer not to answer [file 12916_2025_4272_MOESM1_ESM.docx]

**Table 4**

Clinical characteristics by race and ethnicity subgroups (*N*=1,119) including comparison statistics, *mean*(95% CI) unless otherwise indicated

|  |  | **Clinical variables (baseline)** | | | **Process variables (post-treatment)** | |
| --- | --- | --- | --- | --- | --- | --- |
| **Race and Ethnicity** | Age | Post-traumatic stress symptoms, PCL-6^a^ | Depression symptoms, EPDS^b^ | Perceived social support, MSPSS^c^ | Treatment satisfaction at 3-months post treatment,CSQ-8^d^ | Treatment dosage (no. of completed sessions) |
| 1) Asian (*n*=192) | 34.15  (33.50, 34.79) | 17.88  (17.08, 18.69) | 16.38  (15.82, 16.93) | 5.04  (4.85, 5.22) | 3.38  (3.29, 3.46) | 6.86  (6.58, 7.14) |
| 2) Black (*n*=113) | 31.68  (30.71, 32.65) | 17.64  (16.57, 18.71) | 16.59  (15.93, 17.26) | 4.60  (4.31, 4.83) | 3.36  (3.23, 3.48) | 6.34  (5.87, 6.80) |
| 3) First Nation/Aboriginal (*n*=5) | 31.20  (24.38, 40.02) | 19.8  (12.81, 26.79) | 14.20  (9.95, 18.45) | 4.55  (1.92, 7.19) | 3.55  (2.75, 4.35) | 7.80  (7.24, 8.36) |
| 4) Hawaiian/Pacific Islander  (*n*=4) | 34.75  (26.39, 43.11) | 15.25  (6.89, 23.71) | 13.50  (9.29, 17.71) | 5.33  (2.49, 8.18) | 3.72  (3.20, 4.24) | 6.75  (4.36, 9.14) |
| 5) Hispanic (*n*=93) | 31.82  (30.73, 32.90) | 16.77  (15.48, 18.06) | 15.76  (14.92, 16.60) | 5.09  (4.81, 5.38) | 3.52  (3.40, 3.63) | 6.80  (6.36, 7.23) |
| 6) Middle eastern (*n*=30) | 34.37  (32.58, 36.15) | 19.42  (17.20, 21.66) | 16.93  (15.34, 18.53) | 5.22  (4.74, 5.69) | 3.31  (3.05, 3.57) | 6.83  (6.03, 7.64) |
| 7) Mixed Race (*n*=89) | 32.37  (31.28, 33.46) | 17.93  (17.02, 18.84) | 15.54  (14.69, 16.39) | 5.39  (5.17, 5.62) | 3.51  (3.39, 3.63) | 6.81  (6.36, 7.26) |
| 8) White (*n*=563) | 33.72  (33.34, 34.11) | =B16.13  (15.71, 16.54) | 15.34  (15.04, 15.64) | 5.59  (5.50, 5.67) | 3.39  (3.34, 3.44) | 6.92  (6.76, 7.08) |
| 9) Prefer not to answer (*n*=30) | 34.10  (31.85, 36.35) | 17.03  (14.58, 19.49) | 16.23  (14.53, 17.94) | 5.23  (4.79, 5.66) | 3.35 (3.09, 3.62) | 6.43  (5.55, 7.32) |
| **ANOVA and Post-Hoc Statistics** |  |  |  |  |  |  |
| ANOVA | F(8, 1110)=4.67, *p*<0.001*** | F(8, 1110)=4.06, *p*<0.001*** | F(8, 1110)=2.92, *p*=0.003** | F(8, 1110)=11.93, *p*<0.001*** | F(8, 1110)=1.18, *p*=0.310 | F(8, 1110)=1.24, *p*=0.273 |
| Welch ANOVA | F(8, 39.42)=3.82,  *p* =0.002** | F(8, 39.47)=3.71,  *p*=0.003** | F(8, 39.66)=2.81,  *p*=0.014* | F(8, 39.28)=8.97,  *p*<0.001*** | F(8, 39.84)=1.43,  *p*=0.214 | F(8, 41.93)=3.12,  *p*=0.007** |
| Post-hoc comparisons^e^ | 8>2; 8>5; 1>2; 1>5 | 1>8; 6>8 | 1>8; 6>8; 2>8 | 8>1; 8>2; 8>5; 1>2; 7>2 | - | - |

*Note.* ^a^=Abbreviated PTSD Checklist-6, scored from 6-30. ^b^=Edinburgh Postnatal Depression Scale, scored from 0-30. ^c^=Multidimensional Scale of Perceived Social Support, scored from 1-7. ^d^=Client Satisfaction Questionnaire, scored from 0-4. ^e^=Post-hoc comparisons using Bonferroni correction. **p*<0.05. ***p* < 0.01. ****p* < 0.001.
