# Supplementary material for: Bridges to treatment satisfaction: the roles of trauma, social support, race and ethnicity among perinatal women receiving behavioural activation therapy
Source: BMC Med. 2025 Aug 20;23:489. doi: 10.1186/s12916-025-04272-y (PMC12366383; doi:10.1186/s12916-025-04272-y)
Supplement: Supplementary file 2 — Additional File 2: Table 5. Baseline characteristicsof the overall sample and the qualitative sub-sample [file 12916_2025_4272_MOESM2_ESM.docx]

**Table 5**

Baseline characteristics of overall sample (*N*=1,119) and qualitative sub-sample (*N*=807), *N*(%) unless otherwise indicated

| **Characteristic** | **Sample (*N*=1,119)** | **Qualitative (*N*=807)** |
| --- | --- | --- |
| **Demographic variables (baseline)** |  |  |
| Age, *mean (95% CI)* | 33.35 (33.06, 33.64) | 33.76 (33.44, 34.09) |
| Perinatal period at first session |  |  |
| Pregnant | 556 (49.69) | 398 (49.32) |
| Location |  |  |
| Canada | 751 (67.11) | 613 (75.96) |
| United States | 368 (32.89) | 194 (24.04) |
| Race and ethnicity recategorized |  |  |
| Racial and ethnic minority group* | 526 (47.01) | 368 (45.60) |
| White | 563 (50.31) | 420 (52.04) |
| Race and ethnicity by subgroup |  |  |
| Asian | 192 (17.16) | 135 (16.73) |
| Black | 113 (10.10) | 82 (10.16) |
| First Nations/Aboriginal | 5 (0.45) | 5 (0.62) |
| Hawaiian/Pacific Islander | 4 (0.36) | 4 (0.50) |
| Hispanic | 93 (8.31) | 52 (6.44) |
| Middle Eastern | 30 (2.68) | 24 (2.97) |
| Mixed-race | 89 (7.95) | 66 (8.18) |
| White and European | 563 (50.31) | 420 (52.04) |
| Prefer not to answer | 30 (2.68) | 19 (2.35) |
| Gender identity |  |  |
| Female | 1079 (96.43) | 793 (98.27) |
| Genderqueer/Gender non-conforming | 2 (0.18) | 1 (0.12) |
| Different identity | 1 (0.09) | 0 (0.00) |
| Prefer not to answer | 1 (0.09) | 1 (0.12) |
| Marital status |  |  |
| Married or stable relationship | 966 (86.33) | 706 (87.48) |
| Single | 139 (12.42) | 90 (11.15) |
| Prefer not to answer | 14 (1.25) | 11 (1.36) |
| Education |  |  |
| University (graduate degree) | 799 (71.40) | 590 (73.11) |
| College/Trade school | 186 (16.62) | 133 (16.5) |
| Highschool and below | 125 (11.17) | 80 (9.91) |
| Prefer not to answer | 9 (0.80) | 4 (0.50) |
| Employment |  |  |
| Employed | 565 (50.49) | 420 (52.0) |
| Unemployed | 552 (49.33) | 385 (47.71) |
| Missing | 2 (0.18) | 2 (0.25) |
| Household income (based on postal code) |  |  |
| $0-$39,999 | 307 (27.44) | 101 (12.51) |
| $40,000-$79,999 | 640 (57.19) | 153 (18.96) |
| $80,000+ | 92 (8.22) | 473 (58.6) |
| Prefer not to answer | 80 (7.15) | 80 (9.9) |
| Immigration status (born in country of residence) |  |  |
| Yes | 779 (69.62) | 565 (70.0) |
| No | 334 (29.85) | 239 (29.62) |
| Prefer not to answer | 6 (0.54) | 3 (0.37) |
| Provider type (specialist/non-specialist) |  |  |
| Specialist | 566 (50.58) | 416 (51.55) |
| Delivery type (in-person/telemedicine) |  |  |
| In-person | 232 (20.73) | 629 (77.94) |
| **Clinical variables (baseline),** *mean (95% CI)* |  |  |
| Post-traumatic stress symptoms, PCL-6^a^ | 16.90 (16.59, 17.22) | 17.07 (16.72, 17.43) |
| Depression symptoms, EPDS^b^ | 15.75 (15.52, 15.97) | 15.83 (15.56, 16.09) |
| Perceived social support, MSPSS^c^ | 5.31 (5.24, 5.38) | 5.37 (5.30, 5.46) |
| **Process variables (post-treatment),** *mean (95% CI)* |  |  |
| Treatment Satisfaction at 3-months post-treatment, CSQ-8^d^ | 3.40 (3.37, 3.44) | 3.44 (3.40, 3.49) |
| Treatment dosage (no. of completed sessions) | 6. 82 (6.70, 6.94) | 7.2 (7.08, 7.31) |
| Late completion of 3-month questionnaire, *n(%)* | 6 (0.54) | 4 (0.5) |

*Note.* *Racial and ethnic minority groups=Asian, Black, First Nations/Aboriginal, Hawaiian/Pacific Islander, Hispanic, Middle Eastern and Mixed race. ^a^=Abbreviated PTSD Checklist-6, scored from 6-30. ^b^=Edinburgh Postnatal Depression Scale, scored from 0-30. ^c^=Multidimensional Scale of Perceived Social Support, scored from 1-7. ^d^=Client Satisfaction Questionnaire, scored from 0-4.
